# A molecular epidemiological study of respiratory viruses detected in Japanese children with acute wheezing illness

**DOI:** 10.1186/1471-2334-11-168

**Published:** 2011-06-10

**Authors:** Asako Fujitsuka, Hiroyuki Tsukagoshi, Mika Arakawa, Kazuko Goto-Sugai, Akihide Ryo, Yoshimichi Okayama, Katsumi Mizuta, Atsuyoshi Nishina, Masakazu Yoshizumi, Yoichi Kaburagi, Masahiro Noda, Masato Tashiro, Nobuhiko Okabe, Masaaki Mori, Shumpei Yokota, Hirokazu Kimura

**Affiliations:** 1 Department of Pediatrics, National Hospital Organization Yokohama Medical Center, 3-60-2 Harajuku, Totsuka-ku, Yokohama, Kanagawa 245-8575, Japan; 2 Department of Pediatrics, Yokohama City University Graduate School of Medicine, 3-9 Fukuura, Kanazawa-ku, Yokohama, Kanagawa 236-0004, Japan; 3 Department of Health Science, Gunma Prefectural Institute of Public Health and Environmental Sciences, 378 Kamioki-machi, Maebashi-shi, Gunma 371-0052, Japan; 4 Department of Microbiology, Tochigi Prefectural Institute of Public Health, 2154-13 Shimo-okamoto, Utsunomiya-shi, Tochigi 329-1196, Japan; 5 Department of Molecular Biodefence Research, Yokohama City University Graduate School of Medicine, 3-9 Fukuura, Kanazawa-ku, Yokohama, Kanagawa 236-0004, Japan; 6 Division of Molecular Cell Immunology and Allergology, Advanced Medical Research Center, Nihon University, Graduate School of Medical Science, 30-1 Oyaguchi-kamimachi, Itabashi-ku, Tokyo 173-8610, Japan; 7 Department of Microbiology, Yamagata Prefectural Institute of Public Health, 1-6-6 Toka-machi, Yamagata-shi, Yamagata 990-0031, Japan; 8 Department of Health and Nutrition, Yamagata Prefectural Yonezawa Women's Junior College, 6-15-1Tori-machi, Yonezawa-shi, Yamagata 992-0025, Japan; 9 Department of Virology III, National Institute of Infectious Diseases, 4-7-1 Gakuen, Musashimurayama-shi, Tokyo 208-0011, Japan; 10 Influenza Virus Research Center, National Institute of Infectious Diseases, 4-7-1 Gakuen, Musashimurayama-shi, Tokyo 208-0011, Japan; 11 Infectious Disease Surveillance Center, National Institute of Infectious Diseases, 4-7-1 Gakuen, Musashimurayama-shi, Tokyo 208-0011, Japan

## Abstract

**Background:**

Recent studies strongly suggest that some respiratory viruses are associated with the induction of acute wheezing and/or exacerbation of bronchial asthma. However, molecular epidemiology of these viruses is not exactly known.

**Methods:**

Using PCR technology, we attempted to detect various respiratory viruses from 115 Japanese children. Furthermore, the detected viruses were subjected to homology, pairwise distance, and phylogenetic analysis.

**Results:**

Viruses were detected from 99 (86.1%) patients. Respiratory syncytial virus (RSV) alone and human rhinovirus (HRV) alone were detected in 47 (40.9%) and 36 (31.3%) patients, respectively. Both RSV and HRV were detected in 14 (12.2%) patients. Human metapneumovirus (HMPV) alone and human parainfluenza virus (HPIV) alone were detected in 1 (0.9%) patient each, respectively. Homology and phylogenetic analyses showed that the RSV and HRV strains were classified into genetically diverse species or subgroups. In addition, RSV was the dominant virus detected in patients with no history of wheezing, whereas HRV was dominant in patients with a history of wheezing.

**Conclusions:**

The results suggested that these genetically diverse respiratory viruses, especially RSV and HRV, might be associated with wheezing in Japanese children.

## Background

A range of respiratory viruses are known to cause acute respiratory infections (ARI), including the common cold, bronchiolitis, and pneumonia in humans [1]. The major pathogens are potentially respiratory syncytial virus (RSV), human rhinovirus (HRV), human metapneumovirus (HMPV), human parainfluenza virus (HPIV), enterovirus (EV), influenza viruses (InfV), adenoviruses (AdV), and human bocavirus (HBoV) [2,3]. Respiratory infections by RSV, HRV, and HPIV are implicated in the induction of wheezing and the exacerbation of asthma, although their mechanisms are not clearly known [4]. The prevalence of asthma in developed countries is around 10 to 15% in children, while the prevalence is lower but increasing rapidly in developing countries [5]. Accumulating evidence indicates that the etiology of most cases of asthma, namely virus-induced asthma, is linked to such respiratory virus infections [6-9]. In addition, other epidemiological studies suggest that about 70% of infants have experienced an RSV infection by the age of 1 year, and 100% by the age of 2 years; the host response to the virus varies greatly, but includes upper respiratory tract infections, typical bronchiolitis (with crepitations but no wheeze), and RSV-induced wheezy bronchitis [10,11]. In addition, HRV includes over 100 serotypes and most of these are epidemic, although their epidemiology is not known [12]. Similarly, most children are infected at least once with HPIV early in life, but reinfections occur throughout life [13]. HBoV and HMPV are recently discovered agents of ARI, and these viruses are also associated with the common cold, bronchiolitis, and pneumonia [14]. However, the relationships between these viruses and virus-induced wheezing are not exactly known.

Genetic analyses including sequence and phylogenetic analyses of various viruses enable detailed genetic characterization of these agents. With the use of these methods, detailed molecular epidemiological studies have been reported, even in non-culturable viruses such as HRV species C (HRV-C) or HBoV [15,16]. However, molecular epidemiology of various respiratory viruses with regard to virus-induced asthma is not exactly known. From these backgrounds, we detected various respiratory viruses and performed a molecular epidemiological study of them in Japanese children with acute wheezing illness.

## Methods

### Subjects

One hundred fifteen wheezy Japanese children were enrolled in the present study. A summary of patient data is shown in Table 1. All patients visited the National Hospital Organization Yokohama Medical Center from November 2007 to March 2009. Of these patients, 39 had a history of wheezing, while the other 76 patients had no such history. In addition, 66 patients had viral bronchitis and/or bronchiolitis at consultation. These patients were treated with infusion, oxygen, and β2-agonist or epinephrine nebulization. Informed consent was obtained from the parents of all subjects for the donation of the nasopharyngeal swabs used in this study.

**Table 1:** Subject data in this study

No. of patients	Sex(M/F)	Age(months)	History of wheezing and/or asthma	No. of patients	Sex(M/F)	Age(months)	No. of inpatients and outpatients		No. of cases of bronchitis and/or bronchiolitis	Age(months)	Hospitalization(days)
							inpatients	55*	46*	13.5 ± 21.0	7.1 ± 2.5
			No	76	44/32	16.9 ± 23.9					
115	70/45	20.8 ± 25.7					outpatients	21	7	25.9 ± 28.9	
			
							inpatients	16	8	18.1 ± 17.0	7.1 ± 1.2
			Yes	39	26/13	28.5 ± 27.5*					
							outpatients	23	5	35.7 ± 31.3	

Data are expressed as mean ± SD

M/F: male/female

*p< 0.05

### DNA/RNA extraction, PCR, and sequencing

For viral DNA/RNA extraction, RT-PCR, and sequence analysis, nasopharyngeal swab samples were centrifuged at 3000 × g at 4°C for 15 min, and the supernatants were used for RT-PCR and sequence analysis as described previously [17]. Viral nucleic acid was extracted from the samples using the High Pure Viral Nucleic Acid Kit (Roche Diagnostics, Mannheim, Germany). The reverse transcription reaction mixture was incubated with random hexamers at 42°C for 90 min, followed by incubation at 99°C for 5 min, and then amplification by thermal cycling. The PCR procedures for amplification of various viral genes including RSV [18], HRV [19,20], HMPV [21], HPIV [22], EV [19,20], InfV [23], AdV [24], and HBoV [25] were conducted as previously described. The primers for PCR are shown in Table 2. To avoid carry over and cross-contamination in PCR, the extraction of viral RNA/DNA was conducted in a room physically separate from that used for performing PCR. Furthermore, positive and negative controls were included in all PCR assays. PCR products were determined by electrophoresis on 3% agarose gel. Purification of DNA fragments and nucleotide sequence determination procedures were performed as described previously [17].

**Table 2:** Primers for PCR used in this study

Virus	Primer	Sequence	Reference no.
RSV	ABG490	5'-ATGATTWYCAYTTTGAAGTGTTC-3'	[22]
	F164	5'-GTTATGACACTGGTATACCAA CC-3'	
	AG655	5'-GATCYCAAACCTCAAACCAC-3'	[23]
	BG517	5'-TTYGTTCCCTGTAGTATATGT G-3'	
HRV	EVP4	5'-CTACTTTGGTGTCCGTGTT-3'	[24]
	OL68-1	5'-GGTAAYTTCCACCACCANCC-3'	[25]
HMPV	hMPV-1f	5'-CTTTGGACTTAATGACAGATG-3'	[26]
	hMPV-1r	5'-GTCTTCCTGTGCTAACTTTG-3'	
	hMPV-2f	5'-CATGCCGACCTCTGCAGGAC-3'	[27]
	hMPV-2r	5'-ATGTTGCAYTCYYTTGATTG-3'	
HPIV	PIS1+	5'-CCGGTAATTTCTCATACCTAT G-3'	[28]
	PIS1-	5'-CTTTGGAGCGGAGTTGTTAAG-3'	
	PIS2+	5'-CCATTTACCTAAGTGATGGAAT-3'	
	PIS2-	5'-GCCCTGTTGTATTTGGAAGAGA-3'	
	PIS3+	5'-ACTCCCAAAGTTGATGAAAGAT-3'	
	PIS3-	5'-TAAATCTTGTTGTTGAGATTG-3'	
InfV A	M30F2/08	5'-ATGAGYCTTYTAACCGAGGTCGAAACG-3'	[29]
	M264R3/08	5'-TGGACAAANCGTCTACGCTGCAG-3'	
InfV B	BHA1F1	5'-AATATCCACAAAATGAAG GCAATA-3'	[29]
	BHAR1166	5'-ATCATTCCTTCCCATCCTCCTTCT-3'	
AdV	AdnU-S'2	5'-TTCCCCATGGCNCACAAYAC-3'	[30]
	AdnU-A2	5'-TGCCKRCTCATRGGCTGRAAGTT-3'	
HBoV	188F	5'-GACCTCTGTAAGTACTATTAC-3'	[31]
	542R	5'-CTCTGTGTTGACTGAATACAG-3'	

RSV: respiratory syncytial virus; HRV: human rhinovirus; HMPV: human metapneumovirus; HPIV: human parainfluenza virus; InfV A: influenza virus subtype A; Inf B: influenza virus subtype B; AdV: adenovirus; HBoV: human bocavirus

### Phylogenetic analysis and calculation of pairwise distances

We performed homology and phylogenetic analysis of the *G *gene of RSV, and the *VP4/VP2 *coding region of HRV, because these viruses were the most commonly detected strains. The nucleotide positions of the nucleotide positions of the *G *gene of RSV were 673-912 (240 bp, for subgroup A) or 670-963 (294 bp, for subgroup B), and the *VP4/VP2 *coding region of HRV were 623-1012 (390 bp). We used the CLUSTAL W program on the DNA Data Bank of Japan (DDBJ) homepage http://clustalw.ddbj.nig.ac.jp/top-j.html and TreeExplorer (Version 2.12) http://evolgen.biol.metro-u.ac.jp/TE/. Evolutionary distances were estimated using Kimura's two-parameter method, and phylogenetic trees were constructed using the neighbor-joining (NJ) method [26]. The reliability of the tree was estimated using 1000 bootstrap replications. We selected the reference strains as previously described to construct the phylogenetic trees of RSV and HRV [17,27]. Moreover, we calculated subgroup or species frequency distributions using pairwise genetic distances for each strain, as previously described [17].

### Statistical analysis

Data were analyzed using SPSS software (SPSS for Windows, Version 10.0). All data are expressed as mean ± SD. We performed bivariate analyses using Pearson *χ^2 ^*and Fisher exact tests to compare the prevalence of respiratory viruses and other variables between the study groups. The Student's *t*-test was used to compare mean age in the study group. Statistical significance was set at the level of *p*< 0.05.

### Ethics approval

All samples were collected after written informed consent was obtained from the subjects' parents. The study protocol was approved by the Ethics Committee on Human Research of National Hospital Organization Yokohama Medical Center.

## Results

### Viruses detected in the present subjects

We genetically detected RSV, HRV, HMPV, HPIV, EV, InfV, AdV, and HBoV in samples from 115 Japanese children with acute wheezing (Table 3). RSV alone was detected in 47 patients (40.9%). Among these, subgroups A (RSV-A) and B (RSV-B) were found in 27 and 20 patients, respectively. HRV alone was detected in 36 patients (31.3%), and among these, HRV species A (HRV-A), B (HRV-B), and C (HRV-C) were found in 17, 2, and 17 patients, respectively. Both RSV and HRV were detected in 14 patients (12.2%). Among these, combinations of RSV-A + HRV-A, RSV-A + HRV-B, and RSV-A + HRV-C were found in 5, 1, and 1 patient, respectively. In addition, RSV-B + HRV-A, RSV-B + HRV-B, and RSV-B + HRV-C were found in 2, 1, and 4 patients, respectively. HMPV alone and HPIV alone were detected in 1 patient each, respectively. Finally, no viral genes for RSV, HRV, HMPV, HPIV, EV, InfV, AdV, and HBoV were detected in 16 patients (13.9%). From these data, RSV was revealed to be the dominant species detected in patients with no history of wheezing and/or asthma (38 patients vs. 9 patients, *p*< 0.05), while HRV was dominant in those with a history of wheezing and/or asthma (12 patients vs. 24 patients, *p*< 0.05). These results suggested that RSV and HRV were the major causative agents of acute wheezing in the present study. Moreover, both RSV and HRV were detected in over 10% of patients with acute wheezing.

**Table 3:** Subtypes or species of detected viruses

		No history of wheezing and/or asthma		Having history of wheezing and/or asthma
Virus	No. of strains	Strain name	No. of strains	Strain name

RSV-A	25	RSV/YOK/07/14(AB551036), RSV/YOK/07/22(AB551037), RSV/YOK/07/26(AB551038), RSV/YOK/07/43(AB551039), RSV/YOK/07/51(AB551040), RSV/YOK/07/52(AB551041), RSV/YOK/07/53(AB551042), RSV/YOK/07/66(AB551044), RSV/YOK/08/79(AB551046), RSV/YOK/08/83(AB551047), RSV/YOK/08/113(AB551049), RSV/YOK/08/122(AB551053), RSV/YOK/08/123(AB551054), RSV/YOK/08/125(AB551056), RSV/YOK/08/127(AB551057), RSV/YOK/08/128(AB551058), RSV/YOK/08/130(AB551059), RSV/YOK/08/133(AB551060), RSV/YOK/08/134(AB551061), RSV/YOK/08/141(AB551065), RSV/YOK/08/142(AB551066), RSV/YOK/08/146(AB551069), RSV/YOK/08/148(AB551071), RSV/YOK/08/150(AB551072), RSV/YOK/09/162(AB551075)	2	RSV/YOK/08/73(AB551045), RSV/YOK/08/111(AB551048)

RSV-B	13	RSV/YOK/07/13(AB551078), RSV/YOK/07/16(AB551079), RSV/YOK/07/17(AB551080), RSV/YOK/07/21(AB551081), RSV/YOK/07/32(AB551083), RSV/YOK/07/33(AB551084), RSV/YOK/07/34(AB551085), RSV/YOK/07/38(AB551086), RSV/YOK/07/50(AB551092), RSV/YOK/07/56(AB551093), RSV/YOK/07/60(AB551095), RSV/YOK/07/62(AB551096), RSV/YOK/07/64(AB551097)	7	RSV/YOK/07/4(AB551076), RSV/YOK/07/59(AB551094), RSV/YOK/08/74(AB551102), RSV/YOK/08/80(AB551104), RSV/YOK/08/82(AB551105), RSV/YOK/08/84(AB551106), RSV/YOK/08/88(AB551107)

Subtotal	38		9	

HRV-A	5	HRV/YOK/07/7(AB550346), HRV/YOK/07/61(AB550365), HRV/YOK/08/107(AB550377), HRV/YOK/08/110(AB550379), HRV/YOK/08/112(AB550380)	12	HRV/YOK/07/11(AB550348), HRV/YOK/07/15(AB550350), HRV/YOK/07/19(AB550352), HRV/YOK/07/24(AB550355), HRV/YOK/07/25(AB550356), HRV/YOK/07/36(AB550358), HRV/YOK/08/103(AB550374), HRV/YOK/08/131(AB550389), HRV/YOK/08/153(AB550396), HRV/YOK/08/167(AB550402), HRV/YOK/08/169(AB550403), HRV/YOK/08/171(AB550404)

HRV-B	1	HRV/YOK/08/129(AB550389)	1	HRV/YOK/08/154(AB550397)

HRV-C	6	HRV/YOK/07/5(AB550345), HRV/YOK/07/20(AB550356), HRV/YOK/07/41(AB550368), HRV/YOK/08/100(AB550379), HRV/YOK/09/163(AB550400), HRV/YOK/09/164(AB550401)	11	HRV/YOK/07/2(AB550343), HRV/YOK/07/10(AB550347), HRV/YOK/07/12(AB550349), HRV/YOK/07/18(AB550351), HRV/YOK/07/23(AB550353), HRV/YOK/07/55(AB550371), HRV/YOK/08/86(AB550377), HRV/YOK/08/106(AB550382), HRV/YOK/08/120(AB550386), HRV/YOK/08/126(AB550388), HRV/YOK/08/159(AB550398)

Subtotal	12		24	

RSV-A+HRV-A	2	RSV/YOK/08/116(AB551050) + HRV/YOK/08/116(AB550381), RSV/YOK/08/145(AB551068) + HRV/YOK/08/145(AB550392)	3	RSV/YOK/07/1(AB551033) + HRV/YOK/07/1(AB550342), RSV/YOK/08/117(AB551051) + HRV/YOK/08/117(AB550382), RSV/YOK/08/119(AB551052) + HRV/YOK/08/119(AB550384)

RSV-A+HRV-B		ND	1	RSV/YOK/08/140(AB551064) + HRV/YOK/08/140(AB550392)

RSV-A+HRV-C		ND	1	RSV/YOK/07/3(AB551034) + HRV/YOK/07/3(AB550344)

RSV-B+HRV-A	2	RSV/YOK/07/42(AB551087) + HRV/YOK/07/42(AB550361), RSV/YOK/07/47(AB551090) + HRV/YOK/07/47(AB550363)		ND

RSV-B+HRV-B	1	RSV/YOK/08/118(AB551108) + HRV/YOK/08/118(AB550363)		ND

RSV-B+HRV-C	4	RSV/YOK/07/28(AB551082) + HRV/YOK/07/28(AB550365), RSV/YOK/07/45(AB551088) + HRV/YOK/07/45(AB550405), RSV/YOK/07/46(AB551089) + HRV/YOK/07/46(AB550370), RSV/YOK/07/67(AB551099) + HRV/YOK/07/67(AB550375)		ND

Subtotal	9		5	

HMPV-B2	1	HMPV/YOK/07/44(AB565438)		ND

HPIV-1		ND	1	HPIV/YOK/08/115(AB565748)

Total	60		39	

RSV-A, Respiratory syncytial virus subgroup A;RSV-B, Respiratory syncytial virus subgroup B; HRV-A, Human rhinovirus species A; HRV-B, Human rhinovirus species B; HRV-C, Human rhinovirus species C; HMPV, Human metapneumovirus; HPIV-1, Human parainfluenza virus type 1; ND, Not detected

### Seasonal variations of detected viruses

**Figure 1:**
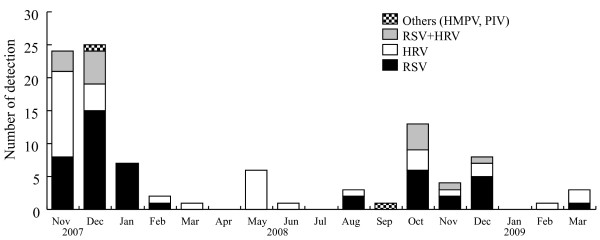
**Seasonal variations of respiratory viruses detected in this study**.

To address relationships between seasonal variations of respiratory viruses and acute wheezing, we showed detected viruses during investigation period as Figure 1. Prevalence of RSV was found from autumn to winter, while prevalence of HRV was found in all season. In addition, both viruses were detected from autumn to winter.

### Homology, phylogenetic analysis, and pairwise distances of RSV and HRV

**Figure 2:**
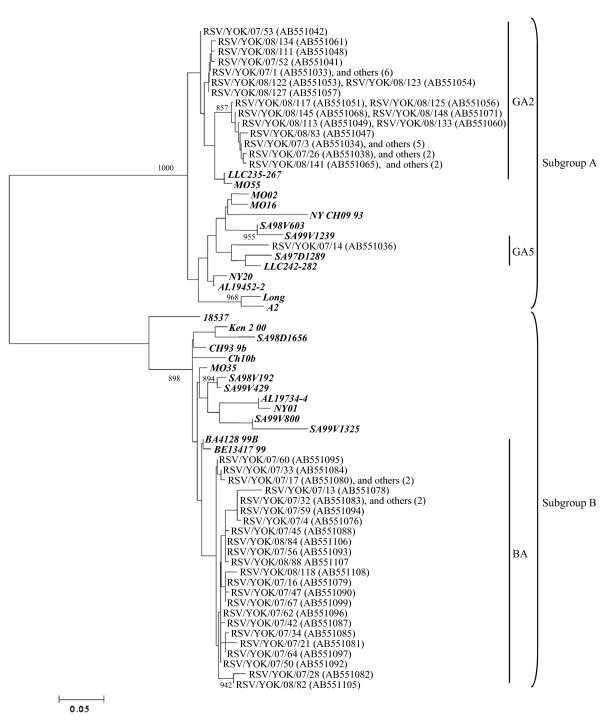
**Phylogenetic analysis of RSV (*G *gene)**. Detailed procedures and conditions of the phylogenetic tree are shown in the text. Numbers in parentheses indicate numbers of strains detected in other patients. Reference strains are shown in bold type. Bars, 0.05 substitutions per nucleotide position. Only bootstrap values more than 85% are shown at branch points.

**Figure 3:**
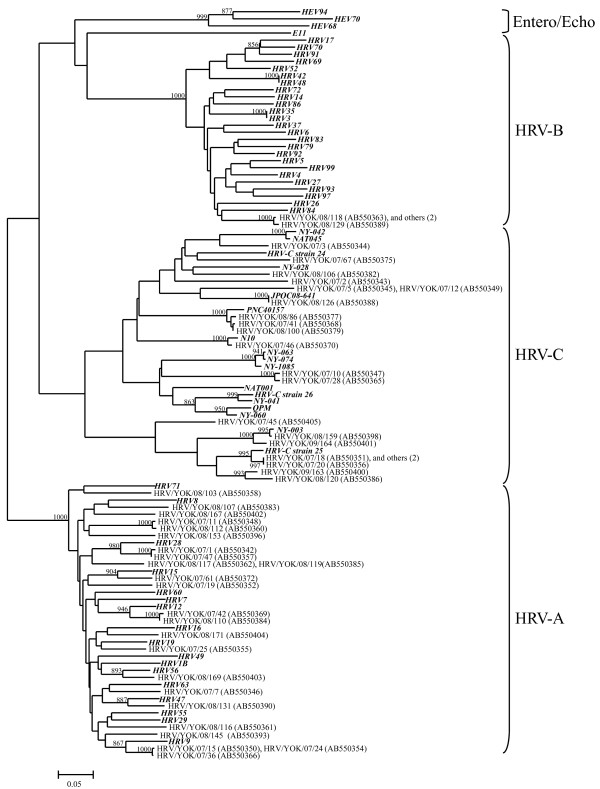
**Phylogenetic analysis of HRV (*VP4/VP2 *coding region)**. Detailed procedures and conditions of the phylogenetic tree are shown in the text. Numbers in parentheses indicate numbers of strains detected in other patients. Reference strains are shown in bold type. Bars, 0.05 substitutions per nucleotide position. Only bootstrap values more than 85% are shown at branch points.

We performed phylogenetic and homology analysis, and calculated the pairwise distances of RSV and HRV in the present cases. The phylogenetic tree based on *G *gene of RSV, and the *VP4/VP2 *coding region of HRV are shown in Figure 2 and 3. The homology and pairwise distances are shown in Table 4. First, the RSV was classified into two subgroups, A and B. In addition, strains belonging to both subgroups were subdivided into three genotypes (GA2, GA5, and BA, Figure 2). HRV was classified into three species: HRV-A, -B, and -C. Strains belonging to these species were subdivided into many clusters in the phylogenetic tree (Figure 3). The homology of RSV-A was relatively high (over 80%), while it was quite low for other viruses and all species of HRV (over 30% divergence). Notably, the genetic diversity of HRV-C was wide (52 to 100%). In addition, the pairwise distances of HRV-A and HRV-C strains are high (over 0.2), while those for RSV-A and RSV-B strains are low. Based on these results it is suggested that acute wheezing-associated HRV has wide genetic diversity.

**Table 4:** Pairwise distances and homology of RSV and HRV strains based on nucleotide sequences

	Homology (%)		Pairwise distance	
Virus	All strains*	Present strains**	All strains*	Present strains**

RSV-A	82.0 - 100	83.5 - 100	0.063 ± 0.043	0.035 ± 0.034
RSV-B	74.2 - 100	92.8 - 100	0.060 ± 0.040	0.029 ± 0.014
HRV-A	66.4 - 100	66.5 - 100	0.202 ± 0.031	0.200 ± 0.038
HRV-B	68.1 - 100	99.5 - 100	0.204 ± 0.039	0.002 ± 0.003
HRV-C	41.0 - 100	52.2 - 100	0.263 ± 0.069	0.254 ± 0.077

Data are expressed as mean ± SD

RSV-A, Respiratory syncytial virus subgroup A

RSV-B, Respiratory syncytial virus subgroup B

HRV-A, Human rhinovirus species A

HRV-B, Human rhinovirus species B

HRV-C, Human rhinovirus species C

* All strains: reference strains plus the strains detected in the present study.

** Present strains: viruses detected in the present study.

## Discussion

We detected and genetically analyzed major ARI viruses including RSV, HRV, HMPV, and HPIV in samples from 115 Japanese children with acute wheezing during a 17-month period (November 2007 and March 2009). These viruses were detected in over 80% of the patients. The dominant viruses were RSV and HRV, and both were detected in over 10% of the patients. In addition, these viruses were confirmed as belonging to various subgroups, genotypes, or species. All three species of HRV detected showed wide genetic diversity (more than 30% divergence). Interestingly, RSV was the dominant species detected in patients with no history of wheezing and/or asthma, while HRV was dominant in patients with a history of wheezing and/or asthma. The results suggested that RSV and HRV were major ARI viruses regarding virus-induced acute wheezing in the present study.

It is suggested that various respiratory viruses such as RSV, HRV, HMPV, HPIV, EV, InfV, AdV, and HBoV are detected in patients with lower respiratory tract infections including bronchiolitis and pneumonia [6,7]. These viruses are also detected in cases of acute wheezing [6,7]. Thus, they may be associated with both lower respiratory tract infection and acute wheezing in children [6,7]. At present, this disease status is recognized by physician and pediatrician as virus-induced asthma [28,29]. It may be important to address the genetic properties of ARI viruses associated with these diseases. However, few studies have been conducted into the genetic analysis of these viruses in acute wheezing. To better understand the relationships between viral properties and acute wheezing, it may be important to genetically analyze ARI viruses detected in the wheezy children. We studied the molecular epidemiology of these respiratory viruses detected in Japanese children with acute wheezing. To the best of our knowledge, the present study is the first to report the detection of RSV, and HRV-A, -B, -C with different genetic characteristics in Japanese children with acute wheezing.

Many studies suggest that RSV is a major candidate as an inducer of acute wheezing [4,10,11] and it may infect all children under the age of 2 years [10,11]. Furthermore, some of these children may develop bronchiolitis and/or pneumonia with acute wheezing [10]. Sugai-Goto *et al*. demonstrated that genotypes and the major genes (*F, G*, and *N*) of RSV isolated from hospitalized children with bronchiolitis or bronchopneumonia accompanied by acute wheezing were not significantly different when compared with RSV strains detected from upper respiratory tract infections [27]. These viruses belong to subgroup A, genotype GA2 and subgroup B, genotype BA [27]. Furthermore, Nakamura *et al*. showed similar genetic data from various acute respiratory infections in Okinawa, Japan [30]. Our findings regarding the properties of *G *gene in the RSV strains detected were comparable with the above-mentioned reports. In contrast, it has been suggested that a specific genotype, GA3 type virus, might be associated with a significantly greater severity of illness [31]. Riccetto *et al*. demonstrated that the severity of illness of RSV infection in infants can be associated with other factors such as body weight and prematurity [32], and any association between the viral properties and pathogenicity of RSV has yet to be elucidated. Another report suggested that host immunity such as TLR4 polymorphism is linked to symptomatic RSV [33]. Thus, both the antigenicity of the viruses and host immune conditions may play important roles in the pathophysiology of severe respiratory infections such as bronchiolitis, pneumonia, and virus-induced asthma [1,34].

For a long time, HRV was simply thought to be causative agents of the mild common cold [12]. In general, this acknowledgement may not be incorrect in non-asthmatic people [35]. However, it is suggested that HRV induces wheezing and exacerbation of symptoms in most asthmatics [12]. However, the molecular epidemiology of each HRV species is not yet known, because HRV is relatively difficult to isolate and detect. Thus, non-culturable HRV-C was only recovered a few years ago. Very recently, Mizuta *et al*. demonstrated that HRV-A isolates showed wide genetic diversity, and some viruses belonging to specific clusters of the phylogenetic tree of HRV-A isolates might be associated with bronchiolitis [17]. In addition, a new study suggested that HRV-C has a stronger link to virus-induced asthma than HRV-A and -B strains [36]. However, our results did not reveal a similar tendency, although the reasons for this are unknown.

In this study, both RSV and HRV were detected from over 10% of patients with acute wheezing. Chung *et al*. demonstrated that both RSV and HRV were detected in 3.9% of Korean children with acute wheezing [37]. Thus, our data and that of other studies may be comparable, although the percentages of virus detection differ. We additionally compared the severity of clinical symptoms between dual virus-detected patients and those in whom RSV or HRV was detected alone. However, there were no significant findings. In addition, RSV, HRV, HMPV, HPIV, EV, InfV, AdV, and HBoV were not detected in over 10% of patients. Although we were unable to explain why, it might be that other respiratory viruses and bacteria were involved.

It should be noted that some respiratory viruses might be detected in healthy children [1,38-40]. As mentioned above, various species of HRV have been relatively frequently detected in healthy children (around 10-20%) [39,40], although RSV was detected less frequently in healthy and asymptomatic persons [40]. Thus, to better understand the etiology of these viruses, it may be important to determine the prevalence of these viruses in healthy children. A limitation of this study is that we did not examine such prevalence in healthy children and instead focused mainly on detailed molecular epidemiological analysis of various respiratory viruses detected in children with acute wheezing. Additional molecular epidemiological studies of viruses detected in wheezy and healthy children would be of value.

In the present study, HMPV and HPIV were detected in samples from the subjects, albeit rarely (each virus was detected in one of only two patients). It is suggested that HMPV and HPIV are also associated with bronchiolitis and bronchopneumonia [41]. However, it is not known how these viruses are linked to the induction of wheezing and exacerbation of asthma [42]. A previous study suggested that sputum from HPIV infection contains tryptase due to activation (degranulation) of mast cells, and this activation may strongly induce an asthmatic attack [43]. Thus, HPIV infection may induce asthmatic conditions [7]. Additional studies regarding the relationships between HPIV and HMPV infection and virus-induced asthma are warranted.

## Conclusions

Our data suggested that both RSV and HRV with various genetic characteristics were associated with acute wheezing illness in Japanese children. In particular, HRV shows widely genetic diversity. Larger studies to examine the detailed genetic characteristics of the various respiratory viruses detected in wheezy and healthy children may be needed.

## Abbreviations

RSV: respiratory syncytial virus; HRV: human rhinovirus; HMPV: human metapneumovirus; HPIV: human parainfluenza virus; EV: enterovirus; InfV: influenza viruses; AdV: adenoviruses; HBoV: human bocavirus; DDBJ: DNA Data Bank of Japan; NJ: neighbor-joining

## Competing interests

The authors declare that they have no competing interests. The authors alone are responsible for the content and writing of the paper.

## Authors' contributions

AF, HK, HT, MT, SY, and NO designed research; MA, KGS, HT, KM, MN, MY, and AN performed research; HT, AF, and MA contributed analytic tools, HK, AR, YO, YK, and MM analyzed data; HK, HT, and YO wrote the paper. All authors read and approved the final manuscript.

## Pre-publication history

The pre-publication history for this paper can be accessed here:

http://www.biomedcentral.com/1471-2334/11/168/prepub
